# ﻿A study of the mealybug genus *Planococcus* Ferris, 1950 from China, with description of a new species (Hemiptera, Coccomorpha, Pseudococcidae)

**DOI:** 10.3897/zookeys.1178.107354

**Published:** 2023-09-04

**Authors:** Jiangtao Zhang, Jun Deng

**Affiliations:** 1 Key Laboratory of National Forestry and Grassland Administration on Forest Ecosystem Protection and Restoration of Poyang Lake Watershed, College of Forestry, Jiangxi Agricultural University, Nanchang, 330045, China Jiangxi Agricultural University Nanchang China; 2 State Key Laboratory of Ecological Pest Control for Fujian and Taiwan Crops, College of Plant Protection, Fujian Agriculture and Forestry University, Fuzhou, 350002, China Fujian Agriculture and Forestry University Fuzhou China

**Keywords:** *Camelliaoleifera*, COI, key, new Chinese record, taxonomy

## Abstract

A study of the mealybug genus *Planococcus* Ferris, 1950 (Hemiptera, Coccomorpha, Pseudococcidae) known from China is presented and 12 species are recognised. Of these, *Planococcuscamelliae* Zhang, **sp. nov.** is described as new to science based on the morphology of the adult female, and *P.bambusifolii* (Takahashi, 1951) is recorded from China for the first time. Molecular analyses based on the mitochondrial gene cytochrome c oxidase subunit I (COI) of the new species and a key to species of the genus *Planococcus* in China are also given.

## ﻿Introduction

The genus *Planococcus* was erected by Ferris (1950) with the citrus mealybug *Dorthesiacitri* Risso, 1813, as the type species, and now comprises 48 species worldwide (García Morales et al. 2016). The genus *Planococcus* includes some well-known pests, such as *Planococcuscitri* (Risso, 1813) on citrus, *P.ficus* (Signoret, 1875) on grapevines, and *P.lilacinus* (Cockerell, 1905) and *P.minor* (Maskell, 1897) on cacao (Cox 1989; Williams 2004), which are often intercepted during quarantine inspections. In China, *P.lilacinus* and *P.minor* became the top two quarantine scale insects intercepted by China inspection and quarantine on imported plants and plant products from 2005 to 2014 (Gu et al. 2015).

Several authors have contributed to the study of *Planococcus* species in China. Ferris (1921) reported that *P.citri* (as *Pseudococcuscitri*) from Taiwan on *Morusalba* (Moraceae). Ezzat and McConnell (1956) described a new species, *Planococcusdorsospinosus* from China, although it was treated as a junior synonym of *P.angkorensis* (Takahashi, 1942) by Williams (2004). Borchsenius (1962a) described three *Planococcus* species, *P.myrsinephilus*, *P.sinensis* and *P.siakwanensis*, from Yunnan, which the first two were regarded as junior synonyms of *P.dorsospinosus* (now *P.angkorensis*) and the last one was treated as junior synonym of *P.kraunhiae* (Kuwana, 1902) (Cox 1989). During the same year, Borchsenius (1962b) reported another mealybug species, *Pedroniaplanococcoides*, from Yunnan, which was transferred to *Planococcus* as *P.planococcoides* by Tang (1992). Later, Tang and Li (1988) reported *Planococcusjuniperus* Tang in Tang & Li from Inner Mongolia. Meanwhile, Tu et al. (1988) reviewed the information of the tribe Planococcini in Taiwan and recorded six *Planococcus* species: *P.angkorensis*, *P.dorsospinosus* (now *P.angkorensis*), *P.citri*, *P.kraunhiae*, *P.lilacinus* and *P.pacificus* Cox, 1981 (now *P.minor*). Subsequently, Cox (1989) provided a systematic revision of the genus *Planococcus* and reported *P.litchi* from China, and Tang (1992) reported *P.lilacinus* as *P.indicus* (Avasthi & Shafee, 1987) from Guangxi. Recently, Pan et al. (2021) reported *P.japonicus* Cox, 1989 from Yunnan and Yuan and Wei (2022) reported *P.vovae* (Nasonov, 1909) from Hebei. To date, there are 10 *Planococcus* species reported from China. Here we describe an additional new species, *P.camelliae* Zhang, sp. nov. and report a new Chinese record, *P.bambusifolii* (Takahashi, 1951).

## ﻿Materials and methods

### ﻿Sampling and morphological identification

Slide-mounted specimens were prepared using the methods of Borchsenius (1950), stained in acid fuchsin, and mounted in Canada balsam. Terminology follows that of Hendricks and Kosztarab (1999) and Williams (2004). Measurements were taken from six specimens. Measurements are given in micrometres (μm) except for the length and width of the body that are in millimetres (mm). Only the known host plants and distribution of each species in China are listed.

Slides of the new species are deposited at the College of Forestry, Jiangxi Agricultural University, Nanchang, China (**CFJAU**) and the Insect Collection of the Southwest Forestry University, Yunnan, China (SWFU).

### ﻿DNA extraction, PCR, and sequencing

DNA extraction, polymerase chain reaction (PCR), and sequencing mainly follow the methods given by Zhang et al. (2018). Two mitochondrial gene cytochrome c oxidase subunit I (COI) sequences of *P.camelliae* were submitted to the NCBI database under the accession numbers OR004347 and OR004348. The sequences of the COI barcode region of *P.citri* (Risso) (KP692647), *P.kraunhiae* (Kuwana) (KP981071), *P.lilacinus* (Cockerell) (KP692654) and *P.minor* (Maskell) (KP692660) were downloaded from GenBank online. Sequences were aligned in BioEdit v. 7.1.3 using ClustalW (Hall 1999). Genetic distances within and between species were calculated using Mega X (Kumar et al. 2018) with the Kimura two-parameter (K2P) model (Kimura 1980).

## ﻿Taxonomy

### ﻿Genus Planococcus

Taxon classificationAnimaliaHemipteraPseudococcidae

Ferris, 1950

D5A5C1F1-F108-58B6-BA2D-F86D3F542F60

Planococcus Ferris, 1950: 164.
Allococcus Ezzat & McConnell, 1956: 13.

#### Diagnosis.

(adapted from Williams 2004). Body of adult female elongate oval to broadly oval. Antennae each with seven or eight segments. Legs well developed, with translucent pores on hind coxa and usually with some on hind tibia, claw without a denticle. Circulus present or absent. Ventral surface of each anal lobe with long apical seta, anal lobe bar and bar seta present. Cisanal setae either longer or shorter than anal ring setae. Anterior and posterior pairs of ostioles present. Marginal cerarii usually 18 pairs, each bearing two conical setae, or some paired flagellate setae, sometimes one or two cerarii of head, each with 3–5 setae; auxiliary setae usually absent, except for anal lobe cerarii. Trilocular pores usually evenly distributed. Oral collar tubular ducts situated on venter, always on abdomen and sometimes on head and thorax; often with indistinct rims when present on dorsum (sometimes termed modified oral collar tubular ducts). Multilocular disc pores rarely present on dorsum, always present on venter of abdomen. Body setae flagellate or short and stiff, sometimes knobbed at apex or almost lanceolate, conical. Discoidal pores present, sometimes larger than trilocular pores.

### ﻿Key to adult females of *Planococcus* known from China (adapted and modified from Williams 2004; Danzig and Gavrilov-Zimin 2010)

**Table:** 

1	Dorsal tubular ducts numerous, located along body margins and in middle areas of body; usually on Cupressaceae	**2**
–	Dorsal tubular ducts, if present, located near cerarii only, or a few in middle part of abdomen, or in small groups along body margins; usually on deciduous trees and shrubs	**3**
2	Cerarii numbering 18 pairs (rarely 14 pairs); circulus present	***P.vovae* (Nasonov)**
–	Cerarii numbering 17 pairs; circulus absent	***P.juniperus* Tang in Tang & Li**
3	Some cerarii on head and thorax, each with more than 2 conical setae, or some cerarii with paired long setae, or 1 conical seta and 1 long flagellate seta	**4**
–	All cerarii on head and thorax, excepted for ocular pair (C_3_), each with 2 conical setae only	**5**
4	All cerarii with conical setae; dorsal setae short and stout	***P.japonicus* Cox**
–	One or more cerarii containing 1 or 2 long and flagellate setae; dorsal setae long and flagellate	***P.lilacinus* (Cockerell)** – in part
5	Multilocular disc pores present on margins of abdominal segments, even if only 1 or 2 present on each segment	**6**
–	Multilocular disc pores absent from margins of abdominal segments entirely, except for an occasional pore	**9**
6	Dorsal oral collar tubular ducts present in groups of 2–5 next to some abdominal cerarii	***P.kraunhiae* (Kuwana)**
–	Dorsal oral collar tubular ducts, if present, located singly next to some abdominal cerarii	**7**
7	Venter of head with 14–35 oral collar tubular ducts and/or thorax with total of 7–35 ducts near 8^th^ pair of cerarii (C_8_); ventral ducts on head and next to 8^th^ pair of cerarii totalling 15–50	***P.citri* (Risso)** – in part
–	Venter of head with 0–13 oral collar tubular ducts and thorax with 0–6 ducts near 8^th^ pair of cerarii (C_8_); ventral ducts on head and next to 8^th^ pair of cerarii totalling 0–18	**8**
8	Ratio of length of hind tibia + tarsus to trochanter + femur 1.10–1.13; multilocular disc pores on posterior edges of abdominal segments IV–VII forming more or less single rows	***P.citri* (Risso)** – in part
–	Ratio of length of hind tibia + tarsus to trochanter + femur 1.04–1.18; multilocular disc pores on posterior edges of abdominal segments IV–VII usually present in double rows	***P.minor* (Maskell)**
9	Ventral multilocular disc pores present on abdominal segment IV	**10**
–	Ventral multilocular disc pores absent from abdominal segment IV	***P.bambusifolii* (Takahashi)**
10	Dorsum with conical setae present, many as large as cerarian setae	**11**
–	Dorsum with flagellate or stout setae present, base of each seta narrower than cerarian setae	**13**
11	Cerarii on dorsal midline, if present, numbering fewer than 7 pairs; translucent pores absent from hind femur	**12**
–	Cerarii on dorsal midline present, numbering 7 or 8 pairs; translucent pores usually present on hind femur	***P.planococcoides* (Borchsenius)**
12	Dorsal setae approx. same size as cerarian setae, mostly with flagellate tips; some dorsal setae on thorax and midline of abdomen present in conspicuous pairs	***P.litchi* Cox**
–	Dorsal setae each usually shorter than a cerarian seta, without flagellate tips; mostly single but occasionally up to 4 conspicuous pairs present	***P.angkorensis* (Takahashi)**
13	Dorsal oral collar tubular ducts present; oral collar tubular present on venter but absent from beneath each postocular cerarius (C_4_)	***P.camelliae* Zhang, sp. nov.**
–	Dorsal oral collar tubular ducts absent; at least 1 ventral oral collar tubular duct usually present beneath each postocular cerarius (C_4_)	***P.lilacinus* (Cockerell)** – in part

### ﻿Descriptions

#### ﻿ Planococcus angkorensis

Taxon classificationAnimaliaHemipteraPseudococcidae

(Takahashi, 1942)

2B690286-7059-5FD1-8EB2-B8DE1AC875FD

Pseudococcus angkorensis Takahashi, 1942: 10.
Planococcus dorsospinosus Ezzat & McConnell, 1956: 75.
Planococcus myrsinephilus Borchsenius, 1962a: 585.
Planococcus sinensis Borchsenius, 1962a: 586.
Planococcus angkorensis : Ali 1970: 89.

##### Host plants.

Anacardiaceae: *Rhus* sp.; Araceae: *Colocasiaesculenta*; Daphniphyllaceae: *Daphniphyllum* sp.; Euphorbiaceae: *Euphorbia* sp.; Fabaceae: *Puerariamontana* (= *P.hirsuta*); Melastomataceae: *Melastoma* sp.; Moraceae: *Ficustinctoria* (= *F.gibbosa*), *Morus* sp.; Myrtaceae: *Psidiumguajava*; Primulaceae: *Myrsineafricana*; Sapindaceae: *Litchi* sp.; Scrophulariaceae: *Buddlejaofficinalis*; Urticaceae: *Oreocnidefrutescens* (= *Boehmeriafrutescens*) (Ezzat and McConnell 1956; Borchsenius 1962a; Tu et al. 1988; Cox 1989; Martin and Lau 2011).

##### Distribution.

Hongkong, Taiwan, Yunnan (Borchsenius 1962a; Tu et al. 1988; Martin and Lau 2011).

##### Remarks.

Good descriptions and illustrations of the adult female can be found in Ezzat and McConnell (1956), Tu et al. (1988), and Williams (2004).

#### ﻿ Planococcus bambusifolii

Taxon classificationAnimaliaHemipteraPseudococcidae

(Takahashi, 1951)

67264686-35AB-588C-9337-4298EDEE09B8

Pseudococcus bambusifolii Takahashi, 1951: 9.
Planococcus bambusifolii : Tang 1992: 367.

##### Material examined.

Guizhou: 2 ♀♀, Southwest Guizhou Autonomous Prefecture, Xingyi City, Maling River Canyon, on bamboo, 7.v.2017, coll. Jiang-tao Zhang & Ming Zhao.

##### Host plant.

Poaceae: bamboo.

##### Distribution.

Guizhou.

##### Remarks.

This is the first record of this mealybug in China. A good description and illustration of the adult female was given by Williams (2004).

#### ﻿ Planococcus camelliae

Taxon classificationAnimaliaHemipteraPseudococcidae

Zhang sp. nov.

1E53595B-24E5-5809-B312-746E382C74F9

https://zoobank.org/D1CFA1AF-A504-4CF3-ACEF-8D9C41CDCD00

Figs 1 , 2 , 3

##### Material examined.

***Holotype***: China: 1 ♀ (mounted singly on a slide), Jiangxi Province, Fuzhou City, Le’an County, Jinzhu she minority Township, Pingxi Village [27°06'N, 115°56'E], on *Camelliaoleifera* (Theaceae), 5.x.2019, coll. Jiang-tao Zhang (CFJAU). ***Paratypes***: 4 ♀♀ (mounted on 4 slides), same data as holotype (2 CFJAU, 2 SWFU); 6 ♀♀ (mounted on 6 slides), same collection and host plant as holotype, 4.viii.2018, coll. Jiang-tao Zhang (4 CFJAU, 2 SWFU).

##### Other material examined.

1 ♀ (mounted on 1 slide), same data as holotype (CFJAU); 8 ♀♀ (mounted on 7 slides), same collection and host plant as holotype, 4.viii.2018, coll. Jiang-tao Zhang (CFJAU).

##### Description.

**Figure 1.**
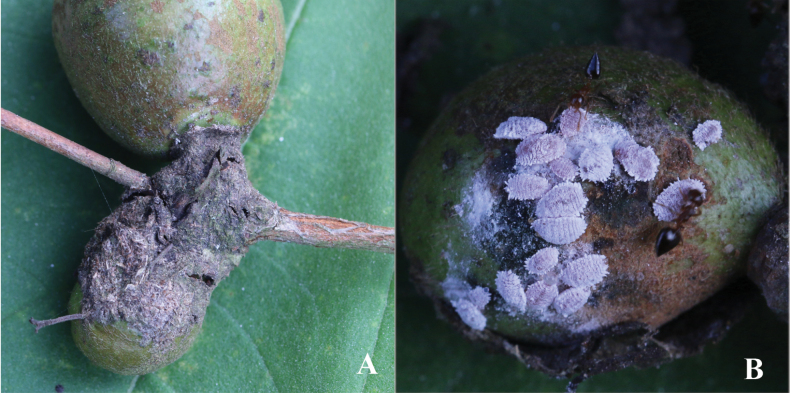
*Planococcuscamelliae* Zhang, sp. nov.: **A** an ant carton that harbours mealybugs inside **B** mealybugs found inside ant carton with tending ants.

In life body oval, covered in white mealy wax, with ~ 18 pairs of short lateral filaments around body margin, found inside ant nests or tents on *Camelliaoleifera* branches and fruits (Fig. 1).

**Figure 2.**
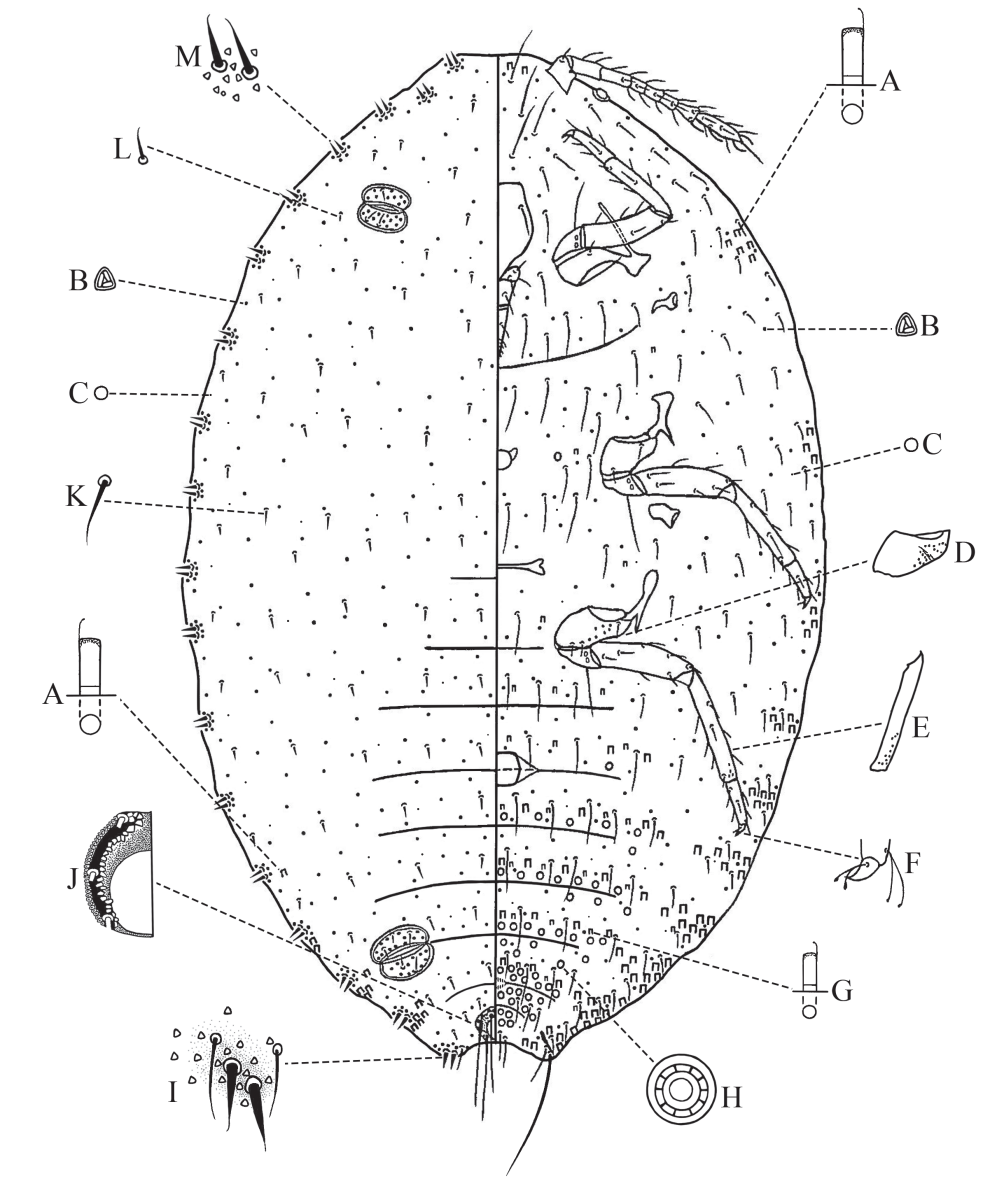
Adult female of *Planococcuscamelliae* Zhang, sp. nov.: **A** Large oral collar tubular duct **B** trilocular pore **C** discoidal pore **D** hind coxa **E** hind tibia **F** claw **G** small oral collar tubular duct **H** multilocular disc pore **I** anal lobe cerarius (C_18_) **J** anal ring **K** large dorsal seta **L** small dorsal seta **M** cerarius on head.

Body of adult female on microscope (Fig. 2) oval, 1.40–3.98 mm long and 0.85–2.39 mm wide. Anal lobes developed, each ventral surface with an apical seta 187–275 μm long, and a well-developed anal lobe bar. Antennae 8-segmented, each 336.5–427.5 μm long, lengths of segments: I 40–50, II 40–52.5, III 42.5–60, IV 20–40, V 22.5–44, VI 30–36.5, VII 37.5–42.5, VIII 87.5–107.5 μm. Clypeolabral shield 142.5–167.5 μm long, 112.5–137.5 μm wide. Labium 131.5–157.5 μm long, 60–75 μm wide. Legs well developed, slender; hind coxa 60–84 μm long, hind trochanter + femur 202.5–277.5 μm long, hind tibia + tarsus 241.5–327.5 μm long; claw 20–25 μm long, both tarsal digitules and claw digitules knobbed, longer than claw. Ratio of lengths of hind tibia + tarsus to hind trochanter + femur 1: 1.11–1.19. Ratio of lengths of hind tibia to tarsus 1: 1.64–2.29. Translucent pores present on hind coxa and tibia. Circulus present, nearly square, 55–112.5 μm long and 50–71.5 μm wide, divided by an intersegmental line. Both anterior and posterior ostioles present, each lip with 7–22 trilocular pores and 1–5 setae. Anal ring 60–85 μm wide, bearing six long setae, each 105–150 μm long. Cerarii numbering 18 pairs. Anal lobe cerarii (C_18_) each bearing two conical setae, each seta 20–24 μm long, accompanied by 2–5 auxiliary setae and 15–25 trilocular pores, situated on a small slightly sclerotised area. Other cerarii, sometimes situated on small prominences, each bearing two conical setae, occasionally only with one conical seta, but ocular pairs (C_3_) sometimes with three conical setae, all cerarian setae conical and with flagellate tips, accompanied by 4–10 trilocular pores. Discoidal pores smaller than the trilocular pores, sparsely but evenly distributed.

**Figure 3.**
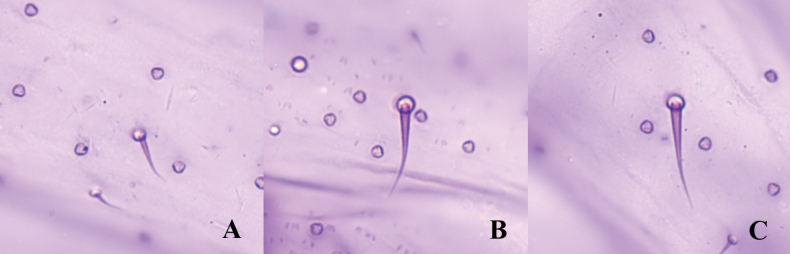
Dorsal setae on *Planococcuscamelliae* Zhang, sp. nov.: **A** small seta **B** large seta with trilocular pore next to setal base **C** large seta without trilocular pore next to setal base.

***Dorsum.*** Setae stout, moderate length and with flagellate tips (Fig. 3), sometimes with one or two trilocular pores next to setal bases, each 20–32.5 μm long. Trilocular pores present, each 3–4 μm in diameter, evenly distributed. Oral collar tubular ducts present and without apparent rims, each 8–10 μm long, 3–4 μm wide, in small marginal groups around posterior abdominal segments, usually 1 duct adjacent to some abdominal cerarii, occasionally also present on median areas of abdominal segments. Multilocular disc pores absent.

***Venter.*** Setae flagellate, longer seta each 75–142.5 μm long. Cisanal setae 50–70 μm long, shorter than anal ring seta. Trilocular pores similar to those on dorsum, evenly distributed. Oral collar tubular ducts of two main sizes: the small type, each 6–7 μm long, 2 μm wide, mainly distributed across middle of abdominal segments II–VII, also a few occurring on median areas of thorax and intermixed with marginal ducts; the large type, similar to those on dorsum, present in transverse rows across abdominal segments III–VII, also in marginal groups around head, thorax, and abdomen, but absent from opposite each postocular cerarius (C_4_). Multilocular disc pores each 8–9 μm in diameter, around vulva, in single or double rows across posterior edges of abdominal segments VII, in single rows across posterior edges of abdominal segments IV–VI, scattered or in single rows across anterior edges of abdominal segments IV–VII or V–VII, sometimes 1–4 pores also on abdominal segment III, a few pores sometimes scattered over median areas of the thorax and head, pores entirely absent from margins of abdominal segments.

##### Host plant.

Theaceae: *Camelliaoleifera*.

##### Distribution.

China (Jiangxi).

##### Remarks.

*Planococcuscamelliae* sp. nov. is similar to *P.kraunhiae* by having oral collar tubular ducts present on dorsum, but it differs from the latter by the following features (condition of *P.kraunhiae* given in parenthesis): (i) multilocular disc pores absent from margins of abdominal segments (present on margins); (ii) single dorsal oral collar tubular ducts usually adjacent to some abdominal cerarii (in groups of 2–5 next to some abdominal cerarii); (iii) dorsal oral collar tubular ducts usually similar in size to the large ducts on venter (usually larger than those on venter). The new species also resembles the type of *P.citri* with oral collar tubular ducts present on dorsum, but it differs from the latter by the following features (condition of *P.citri* given in parenthesis): (i) multilocular disc pores absent from margins of abdominal segments (present on margins); (ii) dorsal setae stout and with flagellate tips (usually flagellate); (iii) dorsal oral collar tubular ducts usually similar in size to the large ducts on venter (if present, usually larger than those on venter).

The pairwise genetic divergences (by K2P distance) in COI among these six specimens (of five species) are listed in Table 1. The K2P distance is 0.00% within *P.camelliae*, 10.44–12.21% between *P.camelliae* and other four *Planococcus* species list. In mealybugs, the interspecific variation in COI is 11.53% (1.96–19.48%) (Wang et al. 2016), and therefore, our molecular data confirms *P.camelliae* as a distinct species.

**Table 1.** Pairwise genetic divergences among *Planococcus* species.

Species	GenBank code	K2P pairwise distances (%) of COI gene				
*P.camelliae*	OR004347	0				
*P.camelliae*	OR004348	0	0			
*P.citri*	KP692647	10.62	10.62			
*P.kraunhiae*	KP981071	12.21	12.21	10.08		
*P.lilacinus*	KP692654	10.44	10.44	7.51	7.90	
*P.minor*	KP692660	11.19	11.19	1.65	9.90	6.97

##### Etymology.

The species epithet is derived from the generic name of its host plant, *Camellia*.

#### ﻿ Planococcus citri

Taxon classificationAnimaliaHemipteraPseudococcidae

(Risso, 1813)

8A129024-529B-5102-AD50-2650B96AC790

Dorthesia citri Risso, 1813: 416.
Coccus tuliparum Bouché, 1844: 301.
Dactylopius alaterni Signoret, 1875: 309.
Dactylopius ceratoniae Signoret, 1875: 311.
Dactylopius cyperi Signoret, 1875: 314.
Dactylopius robiniae Signoret, 1875: 322.
Lecanium phyllococcus Ashmead, 1879: 160.
Dactylopius brevispinus Targioni Tozzetti, 1881: 137.
Dactylopius destructor Comstock, 1881: 342.
Dactylopius secretus Hempel, 1900: 387.
Phenacoccus spiriferus Hempel, 1900: 389.
Pseudococcus citri coleorum Marchal, 1908: 236.
Pseudococcus citri phenacocciformis Brain, 1915: 116.
Planococcus citri : Ferris 1950: 165.
Planococcoides cubanensis Ezzat & McConnell, 1956: 53.
Planococcus citricus Ezzat & McConnell, 1956: 69.
Planococcus cucurbitae Ezzat & McConnell, 1956: 71.

##### Material examined.

Beijing: 2 ♀♀, Changping District, Beiqijia Town, *Aloevera*, 10.xi.2016, coll. Chao-dong Zhu; 5 ♀♀, Beijing world flower Wonderland Park (greenhouse), *Dizygothecaelegantissima*, 7.v.2009, coll. Shan-shan Wang & Wang-mu Deqing; 1 ♀, Tsinghua University (greenhouse), *Codiaeumvariegatum*, 13.iii.2009, coll. Shan-shan Wang & Wang-mu Deqing; 3 ♀♀, Tsinghua University (greenhouse), *Livistonachinensis*, 12.iii.2009, coll. Shan-shan Wang & Wang-mu Deqing; 6 ♀♀, Tsinghua University (greenhouse), *Ficuselastica*, 12.iii.2009, coll. Shan-shan Wang & Wang-mu Deqing; 3 ♀♀, Tsinghua University (greenhouse), host plant unknown, 12.iii.2009, coll. Shan-shan Wang & Wang-mu Deqing; 3 ♀♀, Beijing Forestry University (greenhouse), *Ficusmicrocarpa*, 14.ii.2009, coll. San-an Wu; 4 ♀♀, China Agricultural University (greenhouse), *Asparagussetaceus*, 22.i.2009, coll. Shan-shan Wang; 3 ♀♀, China Agricultural University (greenhouse), *Kalanchoeblossfeldiana*, 22.i.2009, coll. Shan-shan Wang; 17 ♀♀, Beijing Botanical Garden, *Pelargoniumhortorum*, 8.vii.2006, coll. San-an Wu; 3 ♀♀, Beijing Botanical Garden (greenhouse), *Codiaeumvariegatum*, 21.iv.2000, coll. San-an Wu; 2 ♀♀, Beijing Botanical Garden (greenhouse), *Mussaendaesquiroill*, 21.iv.2000, coll. San-an Wu; 5 ♀♀, Beijing Botanical Garden (greenhouse), *Neolamarckiacadamba*, 17.iv.2000, coll. San-an Wu; 4 ♀♀, Beijing Botanical Garden (greenhouse), *Combretumlatifolium*, 25.iii.2000, coll. San-an Wu; 7 ♀♀, Beijing Botanical Garden (greenhouse), *Sedum* sp., 2.xi.1999, coll. San-an Wu. Fujian: 2 ♀♀, Xiamen City, near Yanwu Bridge, *Neriumindicum*, 18.v.2015, coll. Qing-song Zhou & Xu-bo Wang; 8 ♀♀, Zhangzhou City, Pinghe County, *Psidiumguajava*, 22.viii.2011, coll. Ying Guo. Guangdong: 6 ♀♀, Zhanjiang City, *Annonasquamosa*, 26.vii.2010, coll. Yan-biao He. Guangxi: 2 ♀♀, Baise City, Multinational Autonomous County of Longlin, Loushan Road, *Pterocaryastenoptera*, 4.v.2017, coll. Jiang-tao Zhang & Ming Zhao; 2 ♀♀, Baise City, Multinational Autonomous County of Longlin, Yingbin 2 Road, *Artocarpusheterophyllus*, 4.v.2017, coll. Jiang-tao Zhang & Ming Zhao; 2 ♀♀, Baise City, Jingxi City, Chengzhong Road, host plant unknow, 28.iv.2017, coll. Jiang-tao Zhang & Ming Zhao; 2 ♀♀, Wuzhou City, Cangwu County, *Bischofiajavanica*, 5.v.2015, coll. Qing-song Zhou & Xu-bo Wang; 2 ♀♀, Baise City, host plant unknown, coll. Jun Deng, Xu-bo Wang & Xu Zhang. Hainan: 2 ♀♀, Sanya City, *Psidiumguajava*, 10.xii.2011, coll. Hong-wei Luo. Hebei: 14 ♀♀, Cangzhou City, Renqiu City, *Coleusblumei*, 29.ix.2016, coll. Ming-guo Dai. Shanghai: 4 ♀♀, Shanghai Chen Shan Botanical Garden, *Jatrophacurcas*, 9.xii.2010, coll. Ying Xu. Sichuan: 4 ♀♀, Neijiang City, Haozikou Road, *Bischofiajavanica*, 19.vii.2014, coll. Jiang-tao Zhang & Xu-bo Wang; 8 ♀♀, Neijiang City, Haozikou Road, *Erythrinavariegata*, 19.vii.2014, coll. Jiang-tao Zhang & Xu-bo Wang. Xinjiang: 14 ♀♀, Hotan Prefecture (greenhouse), Yutian County, *Neriumoleander*, 12.iii.2010, coll. Ze-zi Ai. Yunnan: 2 ♀♀, Ruili City, Nanmao Lake Park, *Calliandrahaematocephala*, 22.x.2016, coll. Xu-bo Wang & Yao-guang Qin; 1 ♀, Puer City, Lancang Lahu Autonomous County, Menglang Street, *Bischofiajavanica*, 17.x.2016, coll. Xu-bo Wang & Yao-guang Qin; 2 ♀♀, Dai Autonomous Prefecture of Xishuangbanna, Xishuangbanna Tropical Botanical Garden, *Opuntiadillenii*, 23.x.2013, coll. Fang Yu, Jun Deng, Qing-song Zhou & Xu-bo Wang; 3 ♀♀, Dai Autonomous Prefecture of Xishuangbanna, Mengla County, Menglun Town, *Lablabpurpureus*, 23.x.2013, coll. Fang Yu, Jun Deng, Qing-song Zhou & Xu-bo Wang; 2 ♀♀, Dai Autonomous Prefecture of Xishuangbanna, Mengla County, Menglun Town, Cannaindicavar.flava, 23.x.2013, coll. Fang Yu, Jun Deng, Qing-song Zhou & Xu-bo Wang; 2 ♀♀, Dai Autonomous Prefecture of Xishuangbanna, Xishuangbanna Tropical Botanical Garden, *Lucumanervosa*, 22.x.2013, coll. Fang Yu, Jun Deng, Qing-song Zhou & Xu-bo Wang.

##### Host plants.

Acanthaceae: *Pachystachyslutea*; Amaryllidaceae: *Hippeastrumequestre*; Anacardiaceae: *Mangiferaindica*; Annonaceae: *Annonasquamosa*; Apocynaceae: *Adeniumobesum*, *Neriumoleander* (= *N.indicum*); Araliaceae: *Dizygothecaelegantissima*, *Tetrapanaxpapyriferus*; Arecaceae: *Arecacatechu*, *Livistonachinensis*; Asparagaceae: *Asparagussetaceus*; Asphodelaceae: *Aloevera*; Asteraceae: *Bidenschilensis*, *Blumeabalsamifera*, *Erigeroncanadensis*; Cactaceae: *Opuntiadillenii*; Cannaceae: *Cannacoccinea*, *C.flaccida*, *C.indica*, C.indicavar.flava; Combretaceae: *Combretumlatifolium*; Convolvulaceae: *Ipomoeabatatas*; Crassulaceae: *Bryophyllumpinnatum*, *Kalanchoeblossfeldiana*, *Sedum* sp.; Cucurbitaceae: *Cucurbitamoschata*, *Luffacylindrica*; Cyclanthaceae: *Carludovicapalmata*; Dioscoreaceae: *Dioscorea* sp.; Ebenaceae: *Diospyroskaki*, *D.philippensis*; Euphorbiaceae: *Codiaeumvariegatum*, *Euphorbiapulcherrima*, *Jatrophacurcas*, *Macarangatanarius*, *Mallotusjaponicus*; Fabaceae: *Acaciaconfusa*, *Bauhiniapurpurea*, *Calliandrahaematocephala*, *Desmodiumintortum*, *Erythrinavariegata*, *Lablabpurpureus*, *Lespedeza* sp., *Sophoratomentosa*; Geraniaceae: *Pelargoniumhortorum*; Juglandaceae: *Pterocaryastenoptera*; Lamiaceae: *Ajugabracteosa*, *Clerodendrumtrichotomum*, *Coleusblumei*, *Tectonagrandis*; Lauraceae: *Perseagratissima*; Malpighiaceae: *Malpighiaglabra*; Malvaceae: *Theobromacacao*; Marantaceae: *Calatheatubispatha*; Moraceae: *Artocarpusaltilis* (= *A.incisus*), *A.heterophyllus*, *Ficuselastica*, *F.formosana*, *F.microcarpa*, *Morusalba*; Musaceae: *Musasapientum*; Myrtaceae: *Psidiumguajava*; Orchidaceae: *Thrixspermumformosanum*; Pandanaceae: *Pandanusamaryllifolius*; Passifloraceae: *Passifloraedulis*; Phyllanthaceae: *Bischofiajavanica*; Rhizophoraceae: *Kandeliarheedii*; Rosaceae: *Pyrusmalus*; Rubiaceae: *Coffeaarabica*, *Gardeniajasminoides*, *Ixorachinensis*, *Mussaendaesquiroill*, *Neolamarckiacadamba*; Rutaceae: *Citrus* sp., *C.limon*, *C.maxima*, C.medicavar.sarcodactylis, *Clausenalansium*; Sapindaceae: *Dimocarpuslongan*; Sapotaceae: *Lucumanervosa*; Solanaceae: *Solanumaculeatissimum*, *S.diphyllum*, *S.pseudocapsicum*, *S.tuberosum*; Strelitziaceae: *Strelitziareginae*; Theaceae: *Camelliasinensis*; Verbenaceae: *Lantanacamara*, *Verbena* sp. (Martin and Lau 2011; Tsai 2011; Guo et al. 2014; Wang et al. 2016; Wang et al. 2018; Bai et al. 2020).

##### Distribution.

*Planococcuscitri* has a wide distribution in China and it is often difficult to separate from *P.minor* because of the variation in numbers of ventral oral collar tubular ducts. Material examined here is from Beijing, Fujian, Guangdong, Guangxi, Hainan, Hebei, Shanghai, Sichuan, Xinjiang, Yunnan.

##### Remarks.

Good descriptions and illustrations of the adult female can be found in Tu et al. (1988), Cox (1989), and Williams (2004).

#### ﻿ Planococcus japonicus

Taxon classificationAnimaliaHemipteraPseudococcidae

Cox, 1989

173ECE27-AB6F-5141-9221-67FB03B8420D

Planococcus japonicus Cox, 1989: 37.

##### Material examined.

Yunnan: 1 ♀, Dali Bai Autonomous Prefecture, Xiangyun Country, *Osmanthusfragrans*, 10.vii.2021, coll. Hang Chen & Jiang-tao Zhang; 6 ♀♀, Qujing City, Malong District, *Celtis* sp., 7.vii.2019, coll. Jiang-tao Zhang, Kun Hang & Yan Li; 3 ♀♀, Kunming City, Kunming Yunnan West Mountain, *Osmanthusfragrans*, 6.vi.2014, coll. Fu-zhong Wu.

##### Host plants.

Cannabaceae: *Celtis* sp.; Oleaceae: *Osmanthusfragrans* (Pan et al. 2021).

##### Distribution.

Yunnan.

##### Remarks.

Good descriptions and illustrations of the adult female can be found in Cox (1989), Williams (2004), and Pan et al. (2021).

#### ﻿ Planococcus juniperus

Taxon classificationAnimaliaHemipteraPseudococcidae

Tang in Tang & Li, 1988

36C873EF-80C5-548F-9843-F8C591DBF784

Planococcus juniperus Tang in Tang & Li, 1988: 42.
Crisicoccus juniperus : Tang 1992: 352.

##### Host plant.

Cupressaceae: *Juniperusrigida* (Tang and Li 1988).

##### Distribution.

Inner Mongolia (Tang and Li 1988).

##### Remarks.

*Planococcusjuniperus* was transferred to *Crisicoccus* as *C.juniperus* by Tang (1992), but it was subsequently treated as a junior synonym of *P.vovae* (Nasonov) by Danzig and Gavrilov-Zimin (2010). Recently, this mealybug was revived as a valid species by Wu et al. (2023) based on morphological and molecular studies. A good description and illustration of the adult female was given by Tang and Li (1988).

#### ﻿ Planococcus kraunhiae

Taxon classificationAnimaliaHemipteraPseudococcidae

(Kuwana, 1902)

72803C68-CD0D-5779-846F-80DAE43E28CF

Dactylopius kraunhiae Kuwana, 1902: 55.
Planococcus kraunhiae : Ferris 1950: 168.
Planococcus siakwanensis Borchsenius, 1962a: 586.

##### Material examined.

Fujian: 2 ♀♀, Nanping City, Jian’ou City, *Broussonetiapapyrifera*, 5.viii.2019, coll. Jiang-tao Zhang & Kun Huang. Hubei: 11 ♀♀, Jingzhou City, *Broussonetiapapyrifera*, 8.x.2003, coll. San-an Wu. Sichuan: 2 ♀♀, Bazhong City, Pingchang County, Xihuan Street, *Ficusconcinna*, 7.viii.2014, coll. Jiang-tao Zhang & Xu-bo Wang; 9 ♀♀, Nanchong City, Yingshan Country, Waixi Street, *Girardiniadiversifolia*, 3.viii.2014, coll. Jiang-tao Zhang & Xu-bo Wang; 7 ♀♀, Langzhong City, *Broussonetiapapyrifera*, 1.viii.2014, coll. Jiang-tao Zhang & Xu-bo Wang; 2 ♀♀, Mianyang City, Jiangyou City, Taibai Park, *Lagerstroemiaindica*, 29.vii.2014, coll. Jiang-tao Zhang & Xu-bo Wang; 5 ♀♀, Yibin City, Yibin University, *Cercischinensis*, 20.vii.2014, coll. Jiang-tao Zhang & Xu-bo Wang; 1 ♀, Neijiang City, Haozikou Road, *Ficusvirens*, 19.vii.2014, coll. Jiang-tao Zhang & Xu-bo Wang; 2 ♀♀, Nanchong City, Yingshan Country, *Ficusconcinna*, 8.x.2003, coll. Ke Zhang. Yunnan: 2 ♀♀, Kunming City, Shilin Yizu Autonomous County, Changhu Town, *Bidenspilosa*, 11.vii.2019, coll. Jiang-tao Zhang, Kun Huang & Yan Li. Zhejiang: 1 ♀, Lin’an City, Shunxi Town, *Citrusreticulata*, 10.viii.2008, coll. Jin Liu.

##### Host plants.

Asteraceae: *Bidenspilosa*; Casuarinaceae: *Allocasuarinaverticillata* (= *Casuarinastricta*); Dioscoreaceae: *Dioscorea* sp.; Euphorbiaceae: *Macarangatanarius*, *Melanolepismultiglandulosa*; Fabaceae: *Cercischinensis*, *Puerarialobata*; Lythraceae: *Lagerstroemiaindica*; Malvaceae: *Sterculiafoetida*; Moraceae: *Artocarpusnitidus* (= *A.lanceolata*), *Broussonetiapapyrifera*, *Ficusconcinna*, *F.virens*; Myrtaceae: *Psidiumguajava*; Rubiaceae: *Coffeaarabica*; Rutaceae: *Citrusreticulata*; Urticaceae: *Girardiniadiversifolia* (Tsai 2011; Wang et al. 2018).

##### Distribution.

Fujian, Hubei, Sichuan, Taiwan, Yunnan, Zhejiang (Tsai 2011).

##### Remarks.

Good descriptions and illustrations of the adult female can be found in Tu et al. (1988), Cox (1989), and Williams (2004).

#### ﻿ Planococcus lilacinus

Taxon classificationAnimaliaHemipteraPseudococcidae

(Cockerell, 1905)

839A3A6C-75CB-5C1A-B977-5C7455922029

Pseudococcus lilacinus Cockerell, 1905: 128.
Pseudococcus tayabanus Cockerell, 1905: 129.
Dactylopius coffeae Newstead, 1908: 37.
Dactylopius crotonis Green, 1911: 35.
Pseudococcus deceptor Betrem, 1937: 54.
Tylococcus mauritiensis Mamet, 1939: 579.
Planococcus lilacinus : Ferris 1950: 164.
Planococcus indicus Avasthi & Shafee, 1987: 38.

##### Material examined.

Fujian: 2 ♀♀, Zhangzhou City, Zhao’an County, Jinxing Villiage, *Annonasquamosa*, 5.iv.2017, coll. De-yi Yu, Jin-ai Yao & Peng Huang. Guangdong: 5 ♀♀, Heyuan City, Huada Street, *Ficusconcinna*, 10.v.2015, coll. Qing-song Zhou & Xu-bo Wang; 2 ♀♀, Heyuan City, Yanjiang Street, *Ficuselastica*, 10.v.2015, coll. Qing-song Zhou & Xu-bo Wang; 4 ♀♀, Zhongshan City, Yi Ling Road, *Ficusconcinna*, 7.v.2015, coll. Qing-song Zhou & Xu-bo Wang; 5 ♀♀, Guangzhou City, *Bauhiniapurpurea*, 6.xii.2011, coll. Shao-bin Huang. Guangxi: 2 ♀♀, Chongzuo City, Huashan Road, *Lagerstroemiaspeciosa*, 1.v.2017, coll. Jiang-tao Zhang & Ming Zhao; 2 ♀♀, Chongzuo City, Pingxiang City, Pingshan Road, *Ficusconcinna*, 1.v.2017, Jiang-tao Zhang & Ming Zhao; 1 ♀, Chongzuo City, Jiangzhou District, *Bischofiajavanica*, 29.iv.2017, Jiang-tao Zhang & Ming Zhao; 2 ♀♀, Fangchenggang City, Bailu Park, *Lagerstroemiaspeciosa*, 26.iv.2017, Jiang-tao Zhang & Ming Zhao; 5 ♀♀, Hechi City, Chuanshan Town, *Citrus* sp., 26.vii.2015, coll. Jiang-tao Zhang; 6 ♀♀, Beihai City, Dongqu Park, *Broussonetiapapyrifera*, 5.v.2015, coll. Qing-song Zhou & Xu-bo Wang. Hainan: 13 ♀♀, Wanning City, Xinglong Tropical Botanical Garden, *Coffea* sp., 2.v.2009, coll. Guo-qi Zhou. Yunnan: 2 ♀♀, Lancang Lahu Autonomous County, Meng Long Street, *Cassiafistula*, 13.x.2016, coll. Xu-bo Wang & Yao-guang Qin; 2 ♀♀, Dai Autonomous Prefecture of Xishuangbanna, Mengla County, host plant unknown, 13.x.2016, coll. Xu-bo Wang & Yao-guang Qin; 2 ♀♀, Dai Autonomous Prefecture of Xishuangbanna, Xishuangbanna Tropical Botanical Garden, *Capparismasaikai*, 21.x.2013, coll. Jun Deng & Xu-bo Wang. Zhejiang: 8 ♀♀, Wenling City, Daxi Town, *Ficuserecta*, 3.vii.2009, coll. Xiao-xiao Wang.

##### Host plants.

Annonaceae: *Annonareticulata*, *A.squamosa*; Arecaceae: *Roystoneaoleracea*; Cannabaceae: *Celtissinensis*; Capparaceae: *Capparismasaikai*; Combretaceae: *Terminalia* sp.; Ericaceae: *Rhododendron* sp.; Euphorbiaceae: *Aleuritesmoluccanus*, *Codiaeumvariegatum*, *Croton* sp., *Macaranga* sp., *M.sinensis*, *Mallotusjaponicus*, *Melanolepismultiglandulosa*; Fabaceae: *Acaciaconfusa*, *Bauhiniablakeana*, *B.purpurea*, *B.variegata*, *Cassiafistula*, *Dalbergiaodorifera*, *Erythrina* sp., *Tamarindusindica*; Lamiaceae: *Callicarpaformosana*, *Tectonagrandis*; Lythraceae: *Lagerstroemiaindica*, *L.speciosa*; Malvaceae: *Bombaxmalabarica*, *Heritieralittoralis*, *Sterculiafoetida*; Moraceae: *Artocarpusaltilis* (= *A.incisus*), *A.heterophyllus*, *A.xanthocarpus*, *Broussonetiapapyrifera*, *Ficusconcinna*, *F.elastica*, *F.erecta*, *F.microcarpa*; Myricaceae: *Myricarubra*; Myrtaceae: *Psidiumguajava*, *Syzygiumjambos*, *S.samarangense*; Phyllanthaceae: *Bischofiajavanica*, *Bridelia* sp.; Rubiaceae: *Coffea* sp., *C.arabica*, *Gardeniajasminoides*; Rutaceae: *Citrus* sp.; Solanaceae: *Solanumviolaceum* (= *S.indicum*); Tamaricaceae: *Tamarixchinensis* (Tang 1992; Tsai 2011; Ma et al. 2019; Zhang et al. 2019).

##### Distribution.

Fujian, Guangdong, Guangxi, Hainan, Hongkong, Macao, Taiwan, Yunnan, Zhejiang (Tsai 2011; Gu et al. 2015).

##### Remarks.

Good descriptions and illustrations of the adult female can be found in Tu et al. (1988), Cox (1989), and Williams (2004).

#### ﻿ Planococcus litchi

Taxon classificationAnimaliaHemipteraPseudococcidae

Cox, 1989

F2BA80D3-3BA0-5DE5-99E1-73397093E1A0

Planococcus litchi Cox, 1989: 48.

##### Material examined.

Beijing: 12 ♀♀, Institute of Zoology, Chinese Academy of Sciences (indoor), *Radermacherasinica*, 5.v.2014, coll. Xu-bo Wang; 4 ♀♀, Beijing Botanical Garden (greenhouse), *Eriobotryajaponica*, 15.viii.2000, coll. San-an Wu. Guangxi: 1 ♀, Baise City, Multinational Autonomous County of Longlin, Huancheng Road, *Bauhiniablakeana*, 5.v.2017, coll. Jiang-tao Zhang & Ming Zhao; 1 ♀, Baise City, Multinational Autonomous County of Longlin, Huancheng Road, *Dimocarpuslongan*, 5.v.2017, coll. Jiang-tao Zhang & Ming Zhao; 2 ♀♀, Baise City, Jingxi City, Zhongshan Park, *Dimocarpuslongan*, 3.v.2017, coll. Jiang-tao Zhang & Ming Zhao; 2 ♀♀, Fangchenggang City, Fangcheng District, Heti Road, *Dimocarpuslongan*, 28.iv.2017, coll. Jiang-tao Zhang & Ming Zhao. Yuanan: 1 ♀, Jingdong Yi Autonomous County, *Ficusconcinna*, 7.x.2012, coll. Fu-zhong Wu.

##### Host plants.

Bignoniaceae: *Radermacherasinica*; Fabaceae: *Bauhiniablakeana*; Moraceae: *Ficusconcinna*; Rosaceae: *Eriobotryajaponica*; Sapindaceae: *Dimocarpuslongan*, *Litchichinensis* (Cox 1989; Martin and Lau 2011).

##### Distribution.

Beijing, Guangxi, Hongkong, Yunnan (Martin and Lau 2011).

##### Remarks.

Good descriptions and illustrations of the adult female can be found in Cox (1989) and Williams (2004).

#### ﻿ Planococcus minor

Taxon classificationAnimaliaHemipteraPseudococcidae

(Maskell, 1897)

62AEB6DC-36FD-513E-8178-12BAA30BB57C

Dactylopius calceolariae var. minor Maskell, 1897: 322.
Planococcus pacificus Cox, 1981: 48.
Planococcus minor : Cox 1989: 52.
Planococcus psidii Cox, 1989: 62.

##### Material examined.

Beijing: 4 ♀♀, Beijing world flower Wonderland Park (greenhouse), *Saxifraga* sp., 7.v.2009, coll. Shan-shan Wang & Wang-mu Deqing; 4 ♀♀, Beijing world flower Wonderland Park (greenhouse), *Fatsiajaponica*, 7.v.2009, coll. Shan-shan Wang & Wang-mu Deqing; 4 ♀♀, Beijing Botanical Garden (greenhouse), *Codiaeum* sp., 28.ix.2008, coll. Shan-shan Wang & Wang-mu Deqing; 4 ♀♀, Beijing Forestry University (greenhouse), *Sancheziaspeciosa*, 28.ix.2008, coll. Shan-shan Wang & Wang-mu Deqing. Guangdong: 4 ♀♀, Zhanjiang City, *Mangiferaindica*, 12.vi.2010, coll. Yan-biao He. Hainan: 1 ♀, Haikou City, *Mussaendaphilippica*, 6.iv.2015, coll. Bo Cai. Shanghai: 7 ♀♀, *Jatrophacurcas*, 9.xii.2010, coll. Ying Xu. Yunnan: 3 ♀♀, Ruili City, Jiegao, *Albiziajulibrissin*, 23.x.2016, coll. Xu-bo Wang & Yao-guang Qin; 1 ♀, Puer City, Lancang Lahu Autonomous County, *Cannacoccinea*, 18.x.2016, coll. Xu-bo Wang & Yao-guang Qin; 2 ♀♀, Dai Autonomous Prefecture of Xishuangbanna, Mengla County, Menglun town, *Codiaeumvariegatum*, 15.x.2016, coll. Xu-bo Wang & Yao-guang Qin; 2 ♀♀, Dai Autonomous Prefecture of Xishuangbanna, Mengla County, Menglun Town, Costuscomosusvar.bakeri, 15.x.2016, coll. Xu-bo Wang & Yao-guang Qin; 1 ♀, Dai Autonomous Prefecture of Xishuangbanna, Mengla County, *Codiaeumvariegatum*, 13.x.2016, coll. Xu-bo Wang & Yao-guang Qin; 2 ♀♀, Dai Autonomous Prefecture of Xishuangbanna, Jinghong City, Mengbang Road, *Ixorachinensis*, 5.iv.2014, coll. Qing-tao Wu & Xiu-wei Liu; 2 ♀♀, Dai Autonomous Prefecture of Xishuangbanna, Mengla County, Menglun Town, *Fagraeaceilanica*, 23.x.2013, coll. Fang Yu, Jun Deng, Qing-song Zhou & Xu-bo Wang; 3 ♀♀, Jinghong City, Yunnan Institute of Tropical Crops, *Coffea* sp., 24.ix.2012, coll. ?; 10 ♀♀, Jinghong City, Yunnan Institute of Tropical Crops, *Heveabrasiliensis*, 25.viii.2011, coll. Jin-qiang Wang; 4 ♀♀, Dai Autonomous Prefecture of Xishuangbanna, Xishuangbanna Tropical Botanical Garden, *Coffea* sp., 20.x.2008, coll. Kai-ping Ji; 6 ♀♀, Dai Autonomous Prefecture of Xishuangbanna, Xishuangbanna Tropical Botanical Garden, *Coffeaarabica*, 6.iv.2008, coll. Jin Liu, San-an Wu & Shan-shan Wang; 4 ♀♀, Dai Autonomous Prefecture of Xishuangbanna, Xishuangbanna Tropical Botanical Garden, *Bidenspilosa*, 1.iv.2008, coll. Jin Liu, San-an Wu & Shan-shan Wang; 4 ♀♀, Jinghong City, *Heveabrasiliensis*, ix.2007, coll. Zhong-hua Wu; 2 ♀♀, Dai Autonomous Prefecture of Xishuangbanna, Xishuangbanna Tropical Botanical Garden, *Heveabrasiliensis*, 4.vii.2006, coll. Zhong-hua Wu; 6 ♀♀, Jinghong City, *Heveabrasiliensis*, 2006, coll. Zhong-hua Wu.

##### Host plants.

Acanthaceae: *Andrographispaniculata*, *Diclipterachinensis*, *Pachystachyslutea*, *Sancheziaspeciosa*; Altingiaceae: *Liquidambarformosana*; Amaranthaceae: Achyranthesasperavar.indica, *Celosiaargentea*; Amaryllidaceae: *Hippeastrumequestre*; Anacardiaceae: *Mangiferaindica*, *Pistaciachinensis*; Annonaceae: *Annona* sp., *A.squamosa*; Apocynaceae: *Adeniumobesum*, *Asclepiasfruticosa*, *Catharanthusroseus*, *Hoyacarnosa*; Araceae: *Anthuriumscherzerianum*, *Colocasiaesculenta*, *Dieffenbachiapicta*; Araliaceae: *Fatsiajaponica*, *Polysciasfruticosa*, *Scheffleraodorata*; Arecaceae: *Chrysalidocarpuslutescens*, *Hyophorbeamaricaulis*, *Livistonachinensis*; Asparagaceae: *Agaveamericana*, *Cordylinefruticosa*, *Dracaenasurculosa*, *Hostaplantaginea*; Aspleniaceae: *Aspleniumnidus*; Asteraceae: *Artemisiaprinceps*, *Bidenschilensis*, *B.pilosa*, *Chrysanthemumindicum*, *Crassocephalumcrepidioides*, *Dahliahybrida*, *Emiliasonchifolia*, *Gaillardiapulchella*, *Gynurabicolor*, *Lactucasativa*, *Mikaniacordata*, *Wedeliachinensis*; Balsaminaceae: *Impatienswalleriana*; Bignoniaceae: *Pyrostegiavenusta*, *Tabebuiarosea*; Boraginaceae: *Cordiadichotoma*, *Ehretiamicrophylla*, *Messerschmidiaargentea*; Cactaceae: *Mammillariaelongata*; Cannaceae: *Cannacoccinea*, *C.indica*; Convolvulaceae: *Ipomoeaaquatica*, *I.batatas*; Costaceae: Costuscomosusvar.bakeri; Cucurbitaceae: *Cucurbitamoschata*, *Luffacylindrica*; Cyperaceae: *Cyperuspapyrus*; Dioscoreaceae: *Dioscorea* sp.; Dipterocarpaceae: *Parashoreachinensis*; Ebenaceae: *Diospyroskaki*, *D.philippensis*; Euphorbiaceae: *Acalyphahispida*, *Aleuritesfordii*, *Codiaeum* sp., *C.variegatum*, *Euphorbiapulcherrima*, *Heveabrasiliensis*, *Jatrophacurcas*, *Macarangatanarius*, *Mallotusjaponicus*, *Manihotesculenta*, *Melanolepismultiglandulosa*; Fabaceae: *Acaciaconfusa*, *Albiziajulibrissin*, *Arachis* sp., *Bauhiniavariegata*, *Calliandrasurinamensis*, *Cassiaalata*, *Centrosemapubescens*, *Erythrinavariegata*, *Indigoferasuffruticosa*, *Mimosapudica*, *Phaseolusvulgaris*, *Pongamiapinnata*, *Puerarialobata*, *Sophoratomentosa*, *Vignaunguiculatasesquipedalis*; Gentianaceae: *Fagraeaceilanica*; Lamiaceae: *Clerodendrumcyrtophyllum*, *C.paniculatum*, *C.trichotomum*, *Leonurusheterophyllus*, *Menthacanadensis*, *Perillafrutescens*, *Salviaplebeia*; Liliaceae: *Lilium* sp.; Lythraceae: *Punicagranatum*, *Rotala* sp.; Magnoliaceae: *Micheliafigo*; Malpighiaceae: *Malpighiaglabra*; Malvaceae: *Abutilonindicum*, *Bombaxmalabarica*, *Corchoruscapsularis*, *Hibiscusrosa-sinensis*, *H.sabdariffa*, *H.tiliaceus*; Moraceae: *Artocarpusheterophyllus*, *Broussonetiapapyrifera*, *Ficus* sp., *F.benjamina*, *F.elastica*, *F.macrocarpa*, F.superbavar.japonica, *Morusalba*, *M.australis*; Myrtaceae: *Melaleucaleucadendra*, *Psidiumguajava*, *Syzygiumsamarangense*; Orchidaceae: *Cymbidium* sp., *Paphiopedilum* sp.; Oxalidaceae: *Oxaliscorymbosa*; Pandanaceae: *Pandanusamaryllifolius*; Passifloraceae: *Passifloraedulis*; Phyllanthaceae: *Bischofiajavanica*, *Breyniaofficinalis*, *Brideliatomentosa*, *Phyllanthusamarus*; Pinaceae: *Pinusmorrisonicola*; Piperaceae: *Pipernigrum*; Poaceae: *Miscanthusfloridulus*; Portulacaceae: *Portulacapilosa*; Rhamnaceae: *Rhamnaceae*; Rosaceae: *Pyrusserotina*; Rubiaceae: *Coffea* sp., *C.arabica*, *Gardeniajasminoides*, *Ixorachinensis*, *Mussaendaphilippica*; Rutaceae: *Clausenaexcavata*, *C.lansium*, *Citrusmaxima*; Sapindaceae: *Dimocarpuslongan* (= *Euphorialongana*); Saxifragaceae: *Saxifraga* sp.; Solanaceae: *Atropabelladonna*, *Brunfelsiauniflora*, *Daturametel*, *D.suaveolens*, *Solanumdiphyllum*, *S.indicum*, *S.tuberosum*; Theaceae: *Camellia* sp.; Vitaceae: *Ampelopsisbrevipedunculata*; Zingiberaceae: *Hedychiumcoronarium* (Martin and Lau 2011; Tsai 2011; Wang et al. 2016; Lin et al. 2019; Lu et al. 2021).

##### Distribution.

Beijing, Guangdong, Guangxi, Hainan, Hongkong, Macao, Shanghai, Taiwan, Yunnan (Martin and Lau 2011; Tsai 2011; Lu et al. 2021).

##### Remarks.

Good descriptions and illustrations of the adult female can be found in Tu et al. (1988), Cox (1989), and Williams (2004).

#### ﻿ Planococcus planococcoides

Taxon classificationAnimaliaHemipteraPseudococcidae

(Borchsenius, 1962)

834E80CF-A6AB-5265-B519-39827C0D1604

Pedronia planococcoides Borchsenius, 1962b: 235.
Planococcus planococcoides : Tang 1992: 377.
Nipaecoccus planococcoides : Danzig and Gavrilov-Zimin 2015: 482.

##### Material examined.

Yunnan: 1 ♀, Jingdong Yi Autonomous County, *Schimawallichii*, 22.iv.1957, coll. N.S. Borchsenius.

##### Host plants.

Pentaphylacaceae: *Eurya* sp.; Scrophulariaceae: *Buddlejaofficinalis*; Theaceae: *Schimawallichii* (Borchsenius 1962b).

##### Distribution.

Yunnan.

##### Remarks.

*Planococcusplanococcoides* was transferred to *Nipaecoccus* as *N.planococcoides* by Danzig and Gavrilov-Zimin (2015), because it has conical setae on dorsum and additional cerarii along body midline. However, since it has an anal lobe bar present on each anal lobe, marginal cerarii numbering 18 pairs and claw without a denticle, here we follow Tang (1992) and place it under *Planococcus*. A good description and illustration of the adult female was given by Borchsenius (1962b).

#### ﻿ Planococcus vovae

Taxon classificationAnimaliaHemipteraPseudococcidae

(Nasonov, 1909)

3F5C1649-D96D-5C16-9F29-2029D539182C

Coccus gossipifera Rondani, 1874: 43.
Pseudococcus (Dactylopius) vovae Nasonov, 1909: 484.
Pseudococcus inamabilis Hambleton, 1935: 112.
Pseudococcus junipericola Borchsenius, 1949: 116.
Planococcus vovae : Danzig 1980: 168.
Planococcus taigae Danzig, 1986: 19.

##### Material examined.

Beijing: 3 ♀♀, Beijing Forestry University Campus, *Juniperuschinensis*, 9.vi.2022, coll. Yu-ang Li.

##### Host plants.

Cupressaceae: *Juniperuschinensis*, *J.chinensis* ‘Kaizuka’, *J.chinensis* ‘Kaizuka Procumbens’, *J.formosana*, *J.sabina*, *Platycladusorientalis* (Yuan and Wei 2022; Wu et al. 2023).

##### Distribution.

Beijing, Hebei (Yuan and Wei 2022; Wu et al. 2023).

##### Remarks.

Good descriptions and illustrations of the adult female can be found in Cox (1989) and Williams and Granara de Willink (1992).

**Notes.***Planococcusleppulus* (Wu, 2000), which possesses oral rim tubular ducts and cerarii numbering two or three pairs only present on posterior abdominal segments, is not included in this study.

## Supplementary Material

XML Treatment for Planococcus

XML Treatment for Planococcus angkorensis

XML Treatment for Planococcus bambusifolii

XML Treatment for Planococcus camelliae

XML Treatment for Planococcus citri

XML Treatment for Planococcus japonicus

XML Treatment for Planococcus juniperus

XML Treatment for Planococcus kraunhiae

XML Treatment for Planococcus lilacinus

XML Treatment for Planococcus litchi

XML Treatment for Planococcus minor

XML Treatment for Planococcus planococcoides

XML Treatment for Planococcus vovae

## ﻿Additional information

### Author ORCIDs

Jiangtao Zhang https://orcid.org/0000-0002-0007-3918

Jun Deng https://orcid.org/0000-0002-5390-6362

### Data availability

All of the data that support the findings of this study are available in the main text.
